# Supplementary material for: WDR76 is a RAS binding protein that functions as a tumor suppressor via RAS degradation
Source: Nat Commun. 2019 Jan 17;10:295. doi: 10.1038/s41467-018-08230-6 (PMC6336889; doi:10.1038/s41467-018-08230-6)
Supplement: Supplementary file 1 — Supplementary Information [file 41467_2018_8230_MOESM1_ESM.pdf]

Supplementary Information

WDR76 is a RAS binding protein that functions as a tumor suppressor via RAS degradation

Jeong et al.

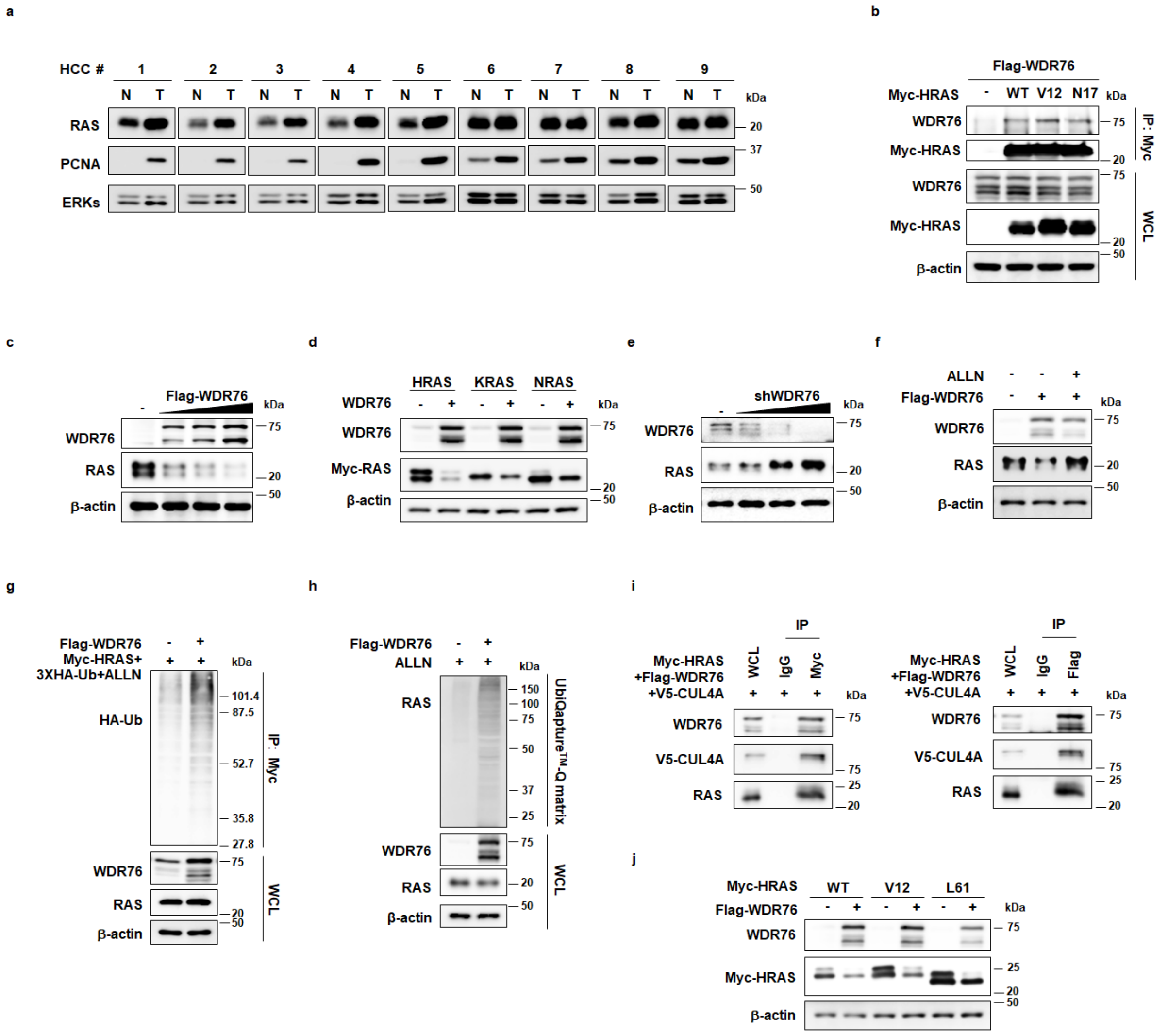

Supplementary Figure 1. WDR76-mediated RAS protein Degradation.

a IB results of RAS, PCNA, and ERKs from HCC tissues (tumor and paired normal). **b** HEK293 cells were transfected with *Myc-HRAS WT, MycH-RAS V12 or MycH-RAS N17* together with *Flag-WDR76* construct then treated with ALLN. WCLs were immunoprecipitated with anti-Myc antibody. **c** HEK293 cells were transfected with *Flag-WDR76* construct. **d** HEK293 cells were transfected with *Myc-HRAS, Myc-KRAS or Myc-NRAS* together with *Flag-WDR76* construct. **e**, HEK293 cells were transfected with *shWDR76* construct. **f** HEK293 cells were transfected with *Flag-WDR76* construct and then

treated with ALLN where indicated. **g, h** HEK293 cells were transfected with indicated vectors and then treated with ALLN. WCLs were then immunoprecipitated with an anti-Myc antibody under denaturing conditions **(g)**. Ubiquitin-conjugated proteins were captured from the lysate using an UbiQapture-Q kit and blotted with anti-RAS antibody **(h)**. **i** HEK293 cells were transfected with *Myc-HRAS, Flag-WDR76* together with *V5-CUL4A* construct then treated with ALLN. WCLs were immunoprecipitated with indicated antibodies. **j** HEK293 cells were transfected with *Myc-HRAS WT, Myc-HRAS V12 or Myc-HRAS L61* together with *Flag-WDR76* construct.

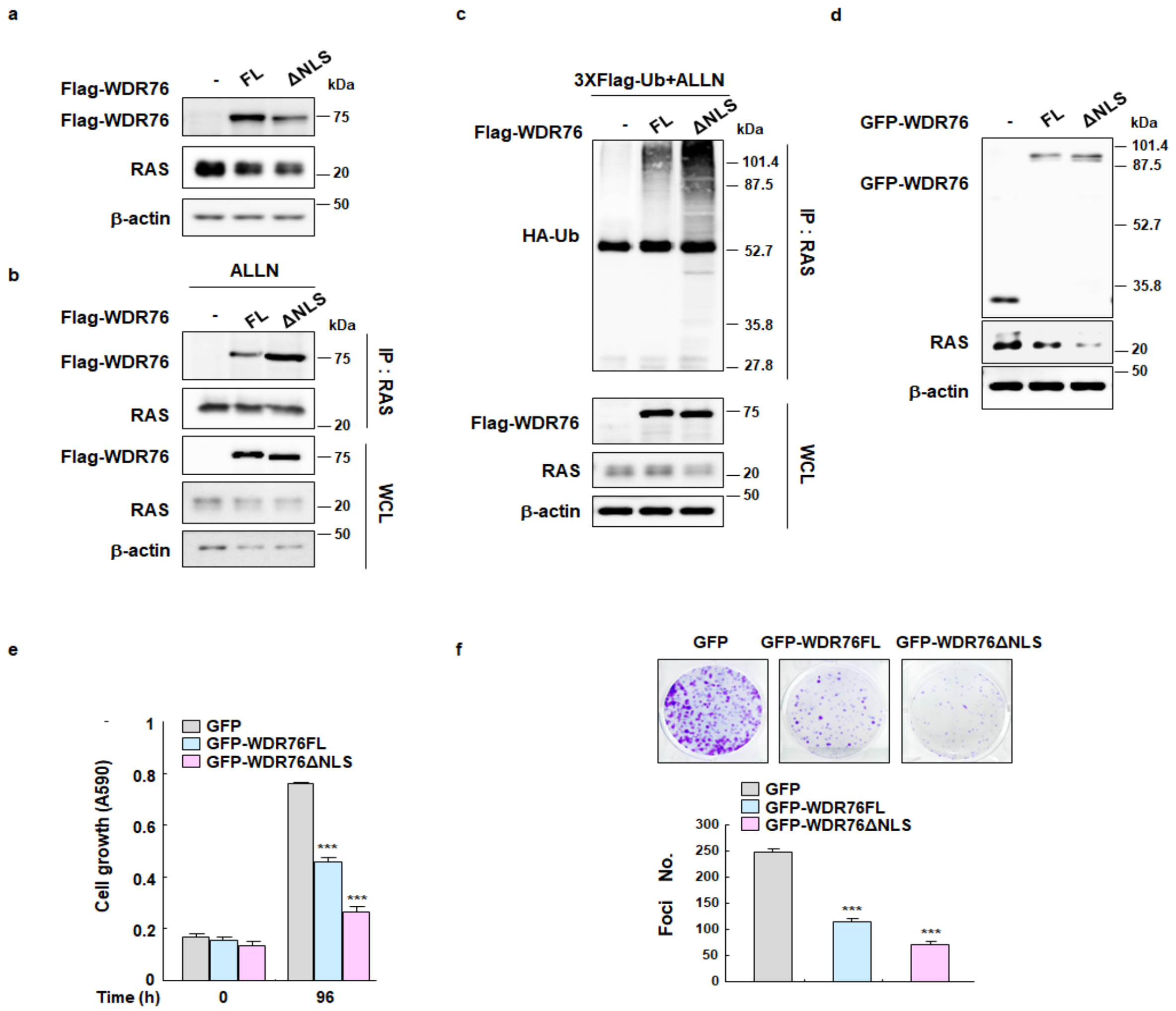

Supplementary Figure 2. Effects of WDR76 on cell proliferation and transformation through RAS degradation.

a-c HEK293 cells were transfected with indicated vectors and analyzed **(a)** or treated with ALLN **(b, c)**. WCLs were immunoprecipitated with anti-RAS antibody **(b, c)**. **d-f**, SK-hep1 cells stably expressing GFP, GFP-WDR76FL, or GFP-WDR76ΔNLS were cultured and analyzed by IB **(d)**, cell growth was assessed by MTT assay **(e)**, and foci formation assays were performed to detect cellular transformation **(f)**. Data are presented as the mean ± SD. (*n* = 3 biological replicates). Two-sided student's *t* test, $^{***}P < 0.001$.

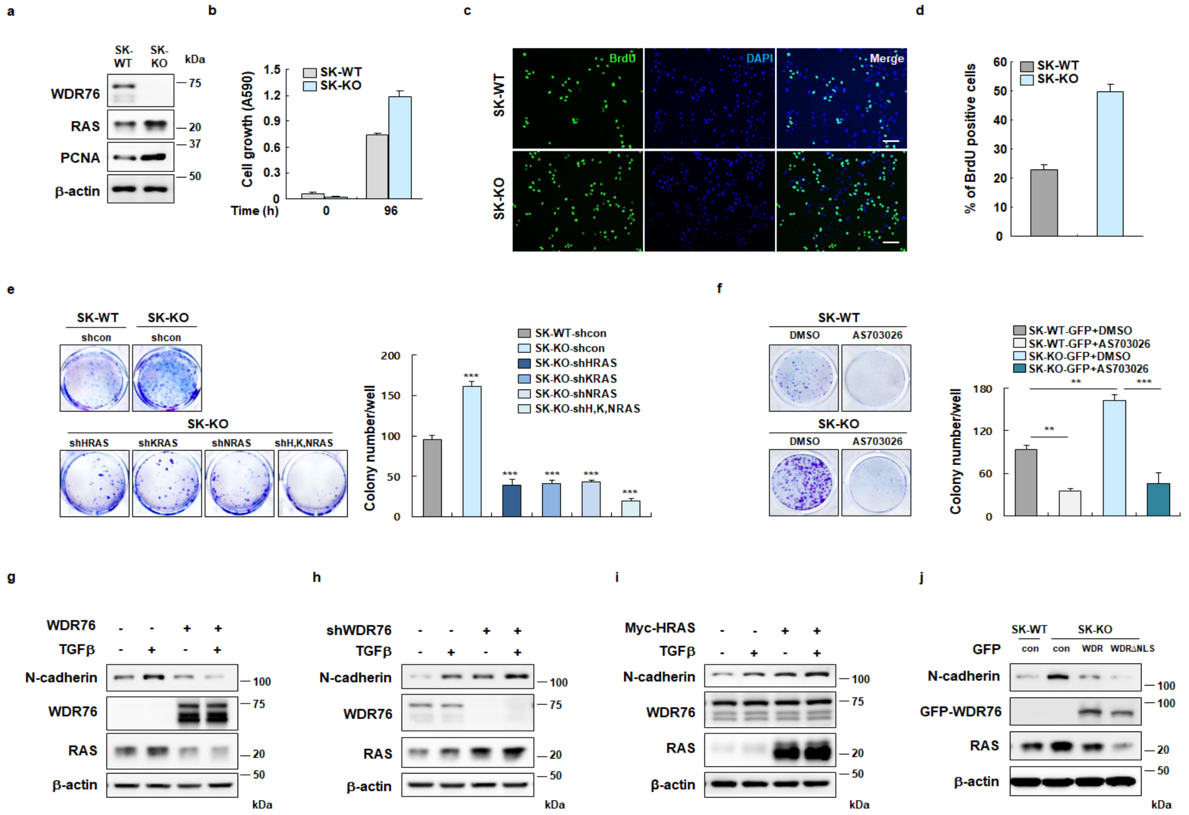

Supplementary Figure 3. Role of WDR76 in the cell proliferation, transformation, and TGFβ-induced EMT. **a-d** IB analyses **(a)**, MTT assays **(b)**, and BrdU (5-bromo-2'-deoxyuridine) incorporation assays **(c, d)** of the SK-WT or SK-KO cells. Scale bars, 100 µm. **e, f** SK-WT and SK-KO cells were transduced with indicated combinations of lentivirus encoding RAS shRNA **(e)** or were treated with DMSO or AS703026 as indicated **(f)**. Cells were applied to foci formation assays and colonies were counted and plotted (right panel) as mean ± SD. (n = 3 biological replicates). Two-sided student's t test, $^{**}P < 0.01$, $^{***}P < 0.001$. **g-i** SK-hep1 cells were transfected with *WDR76* **(g)**, *shWDR76* construct **(h)**, or HRAS **(i)** and treated with TGFβ as indicated. **j**, SK-WT-GFP, SK-KO-GFP, SK-KO-GFP-WDR76FL, or SK-KO-GFP-WDR76ΔNLS cells were cultured. IBs were performed with indicated antibodies.

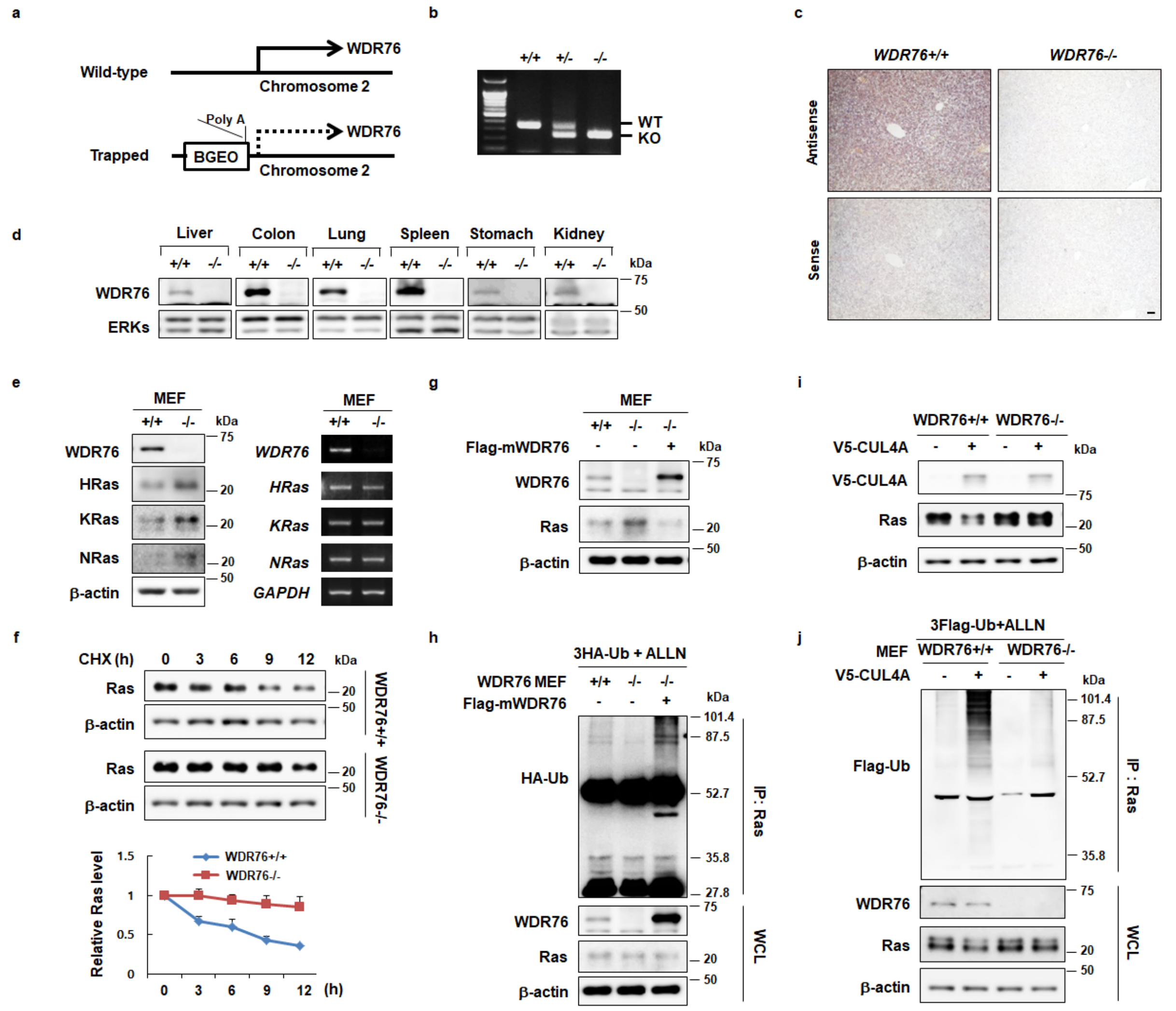

Supplementary Figure 4. Generation of the *WDR76* knockout mouse and stabilization of Ras in *WDR76*$^{-/-}$ MEFs.

a Schematic presentation of the gene trap strategy. The WDR76 gene is located on mouse chromosome 2. **b** PCR analysis of genomic DNA isolated from tails of WT (+/+), heterozygous (+/-), and homozygous WDR76 KO (-/-) mice. **c** *In situ* hybridization was performed for WDR76 in liver sections from 2-week-old *WDR76*$^{+/+}$ and *WDR76*$^{-/-}$ mice. Scale bars, 100 µm. **d** IB examination of WDR76 from the liver, colon, lung, spleen, stomach, and kidney tissue lysates of 2-week-old *WDR76*$^{+/+}$ and *WDR76*$^{-/-}$ mice. **e** IB (left panel) and

RT-PCR (right panel) analyses of *WDR76*$^{+/+}$ and *WDR76*$^{-/-}$ MEFs. f The levels of Ras at indicated time points after CHX treatment were determined by IB of the WCLs, and were quantified with β-actin as a loading control. The bottom panel shows plotted results for the amounts of Ras at each time point relative to the level at time 0. Data are presented as the mean ± SD (*n* = 3 biological replicates). **g, h** *WDR76*$^{+/+}$ MEFs, *WDR76*$^{-/-}$ MEFs or *WDR76* reintroduced *WDR76*$^{-/-}$ MEFs were analyzed **(g)**, or were transfected with 3XHA-Ub and then treated with ALLN **(h)**. WCLs were immunoprecipitated with antibody recognizing Ras. **i, j** *WDR76*$^{+/+}$ MEFs or *WDR76*$^{-/-}$ MEFs were transfected with *V5-CUL4A* together with *3XFlag-Ub* construct then treated with ALLN as indicated **(j)**. WCLs were immunoprecipitated with anti-Ras antibody.

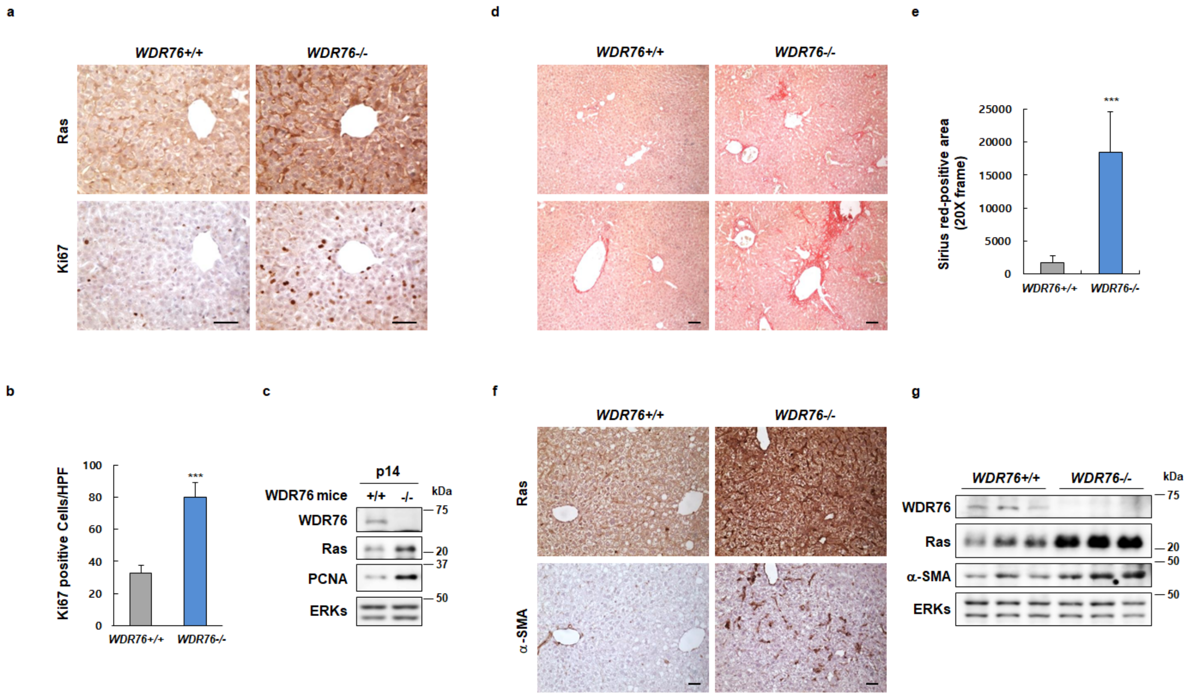

Supplementary Figure 5. Increased hepatic fibrosis in aged *WDR76* knockout mice.

a, b Representative images of Ras, and Ki67 staining in liver sections from 2-week-old *WDR76*$^{+/+}$ or *WDR76*$^{-/-}$ mice **(a)**. Bar graph shows the results for the numbers of Ki67 positive cells per 10 high power field (HPF) **(b)**. Data are presented as the mean ± SD. Two-sided student's t test, $^{***}P < 0.001$. **c** Liver tissues from 2-week-old *WDR76*$^{+/+}$ or *WDR76*$^{-/-}$ mice were subjected to immunoblot to detect indicated proteins. **d, e** Sirius red staining **(d)** and Sirius red-positive area **(e)** of liver tissues from 1-year-old *WDR76*$^{+/+}$ and *WDR76*$^{-/-}$ mice. Data are presented as the mean ± SD (n = 10 biological replicates). Two-sided student's t test, $^{***}P < 0.001$. **f, g** IHC **(f)** and IB **(g)** analyses of liver tissues from 1-year-old *WDR76*$^{+/+}$ and *WDR76*$^{-/-}$ mice. Scale bars, 100 μm.

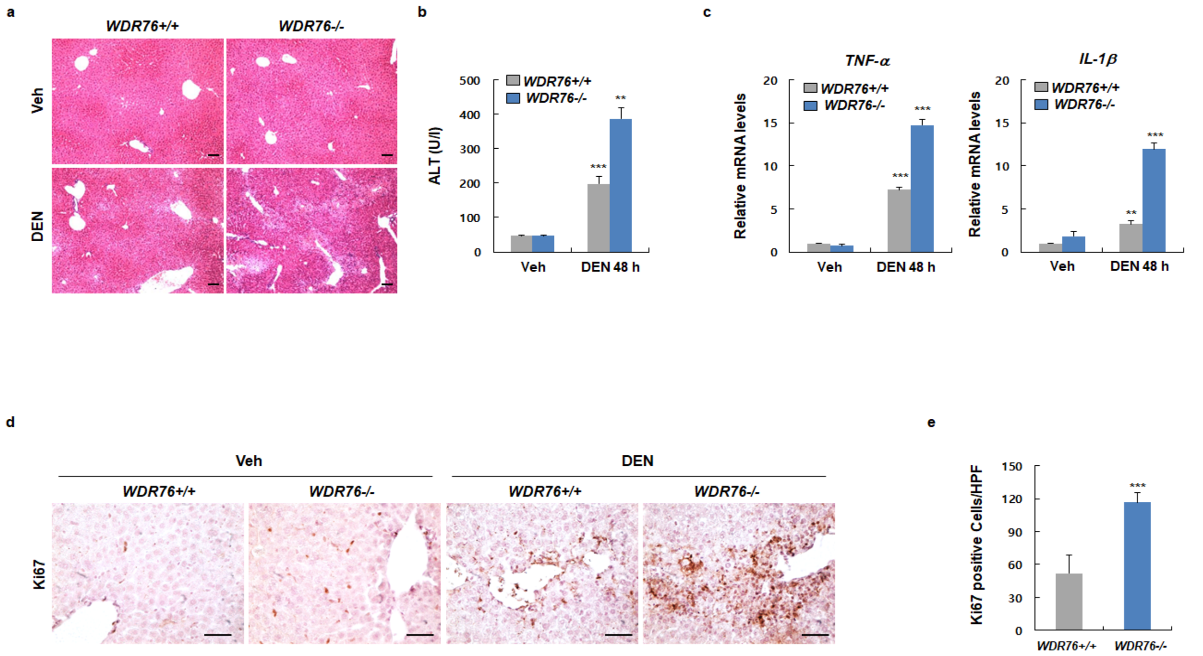

Supplementary Fig. 6 *WDR76* knockout mouse livers are more sensitive to DEN-induced acute liver injury. a-c H&E staining of liver sections **(a)**, ALT levels **(b)**, and proinflammatory gene expression **(c)** of $WDR76^{+/+}$ and $WDR76^{-/-}$ mice that were treated with either vehicle (0.9% NaCl) or DEN at 100 mg/kg body weight. Scale bars, 200 µm. Data are presented as the mean ± SD. (n = 3 biological replicates). Two-sided student's t test, $^{**}P < 0.01$, $^{***}P < 0.001$. **d, e** Representative Ki67 staining of liver sections from $WDR76^{+/+}$ and $WDR76^{-/-}$ mice **(d)** and results of statistical analysis of the numbers of Ki67-positive cells per 10 HPF **(e)**. Data are presented as the mean ± SD. Two-sided student's t test, $^{***}P < 0.001$. Scale bars, 100 µm.

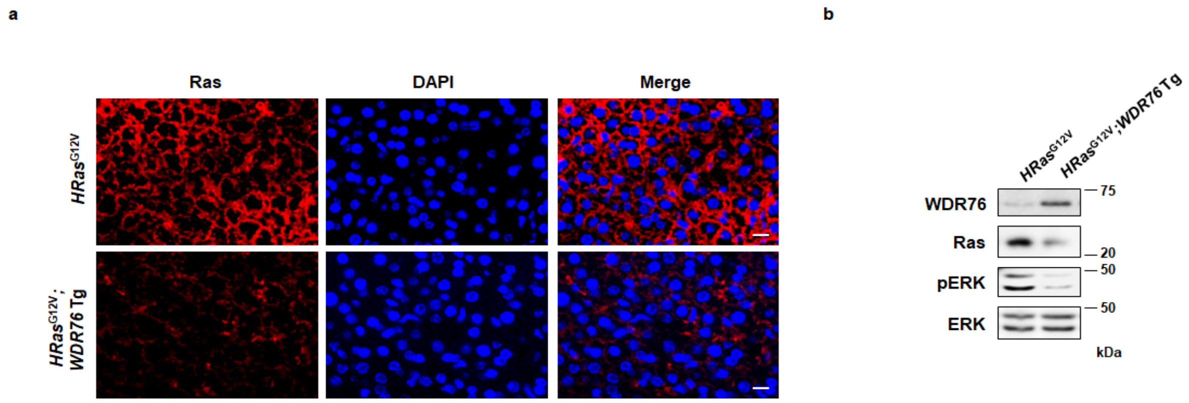

Supplementary Fig. 7 Effect of WDR76 on the stability of RAS in livers from *HRas*^{G12V} mice. a, b IHC **(a)** and IB **(b)** analyses of liver samples from 6-week-old *HRas*^{G12V} or *HRas*^{G12V};*WDR76*Tg mice. Scale bar, 20 µm.

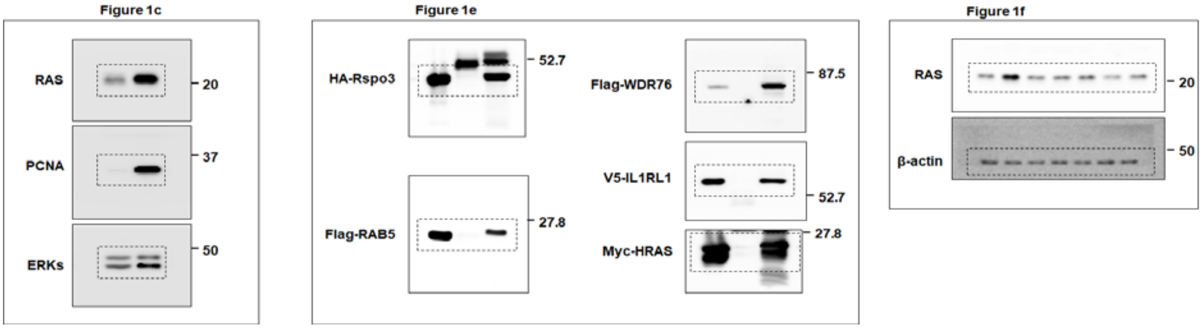

Supplementary Fig. 8 Uncropped blots for Figure 1

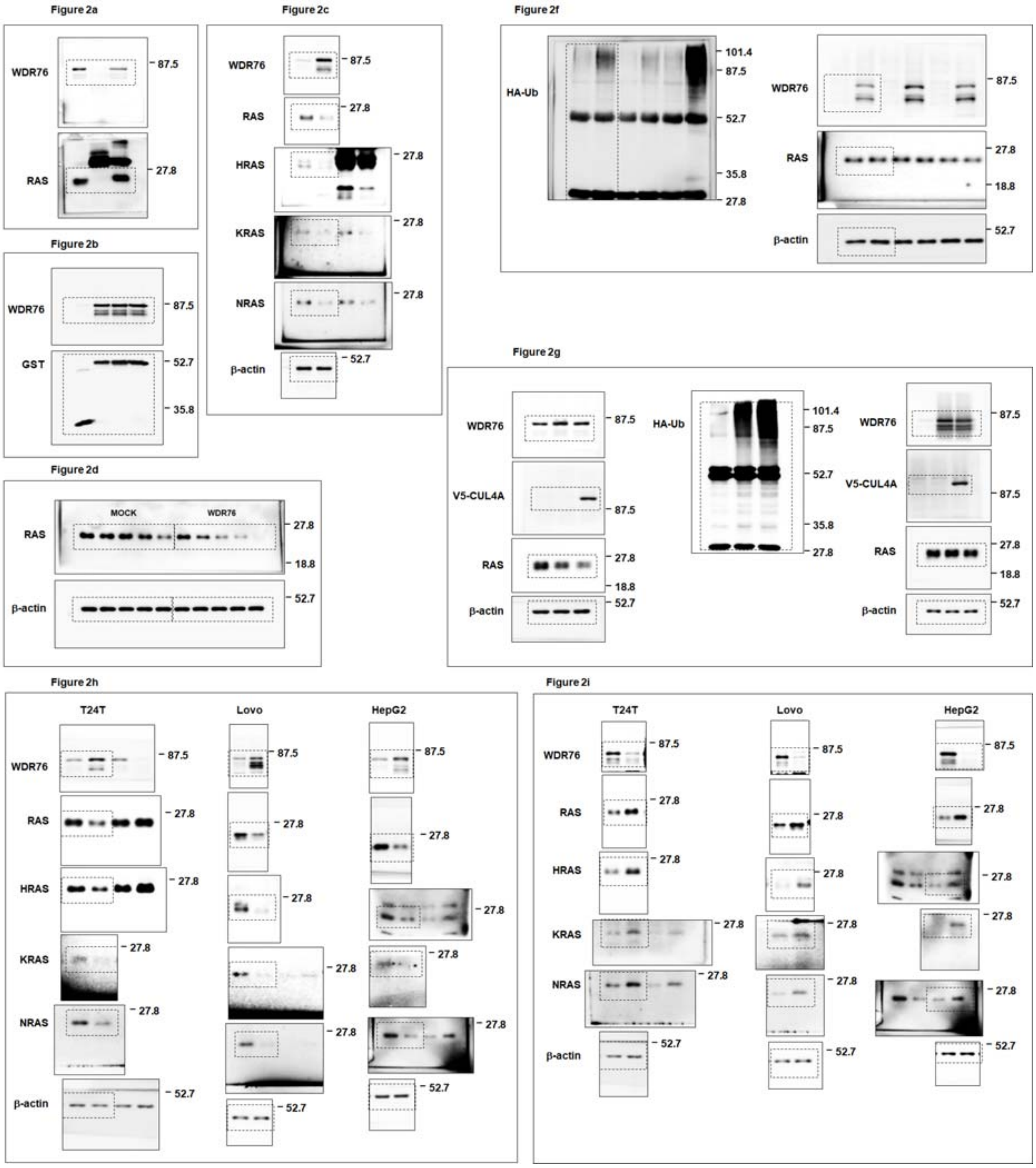

Supplementary Fig. 9 Uncropped blots for Figure 2

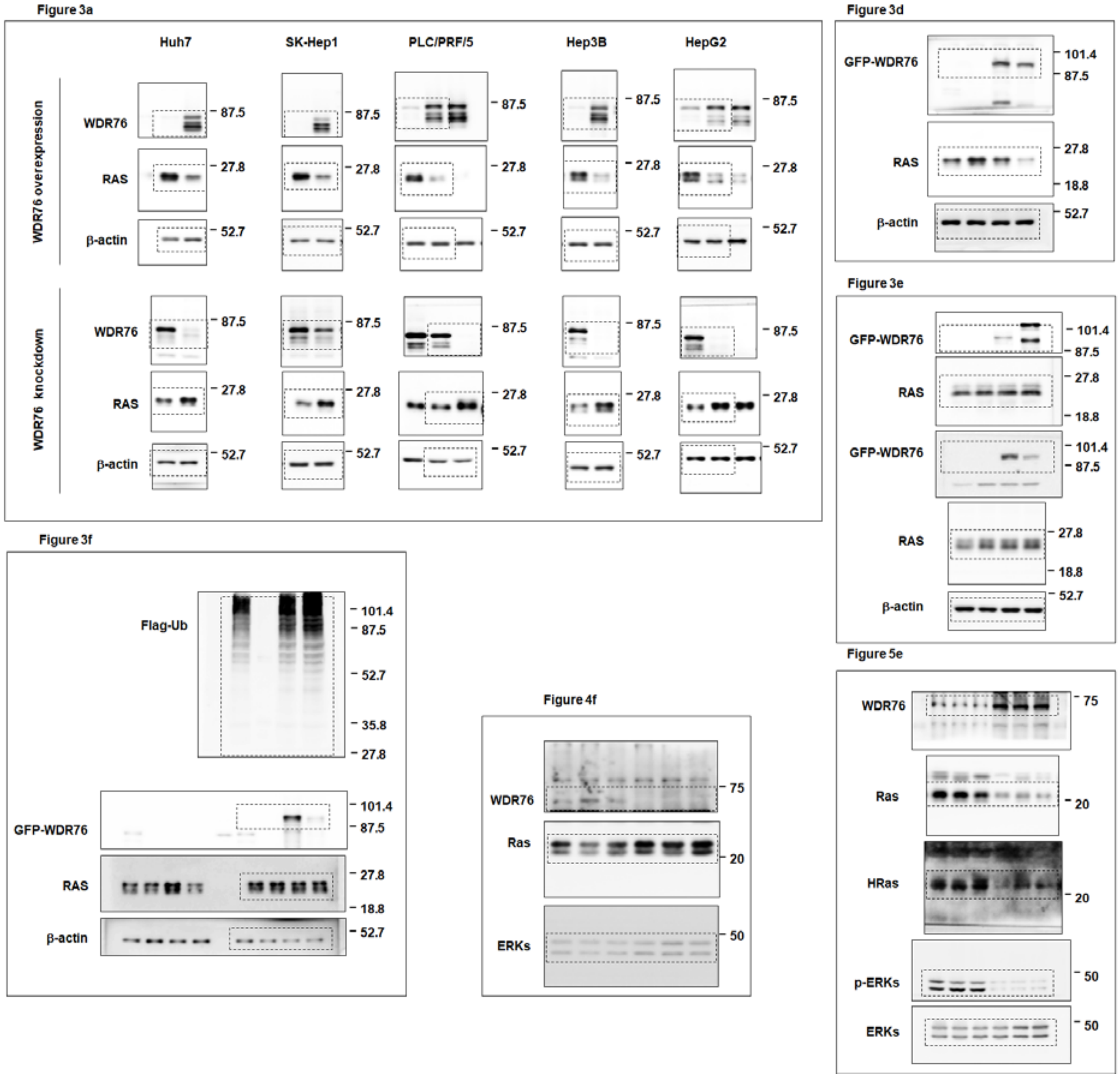

Supplementary Fig. 10 Uncropped blots for Figure 3-5

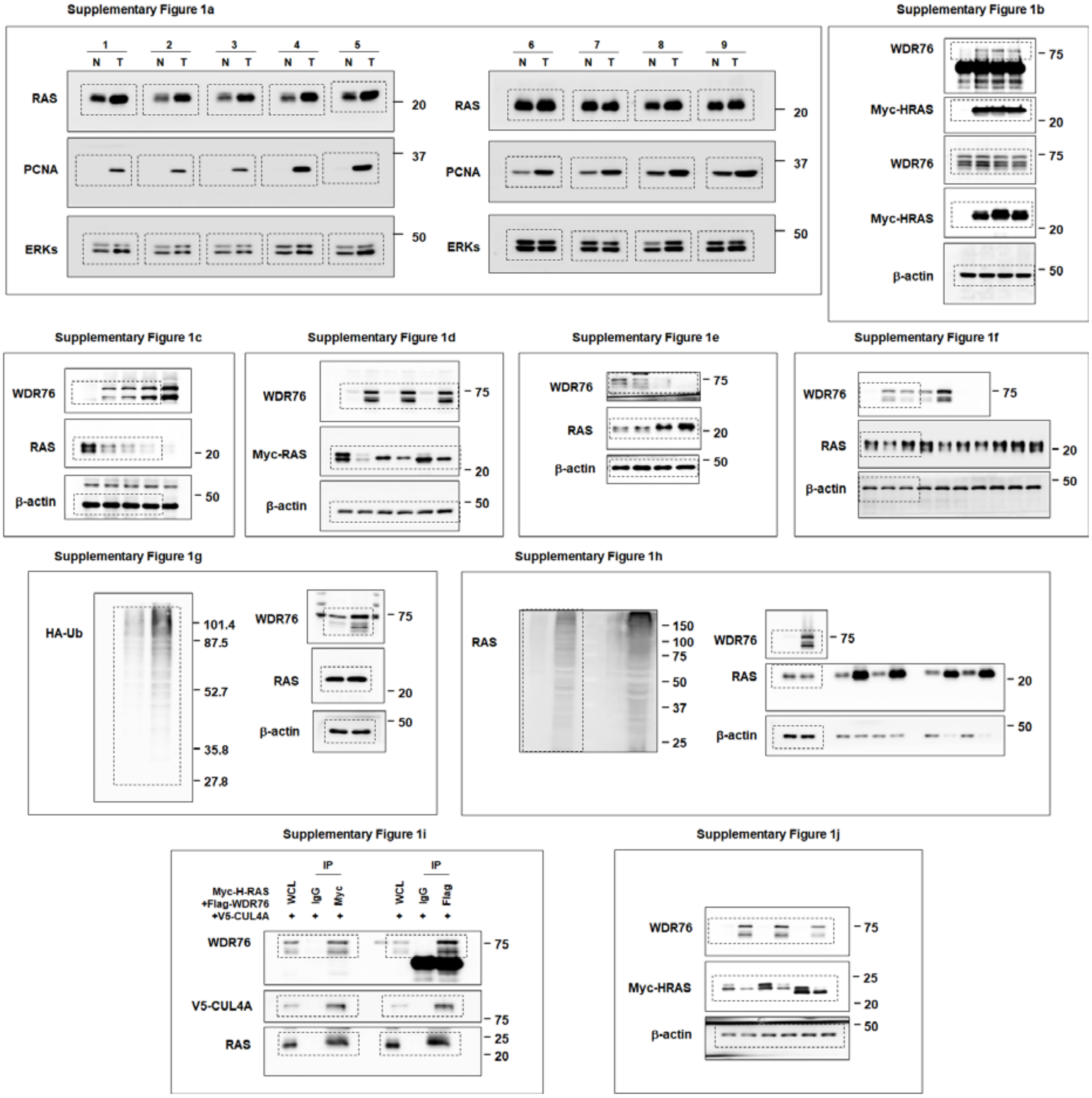

Supplementary Fig. 11 Uncropped blots for Supplementary Figure 1

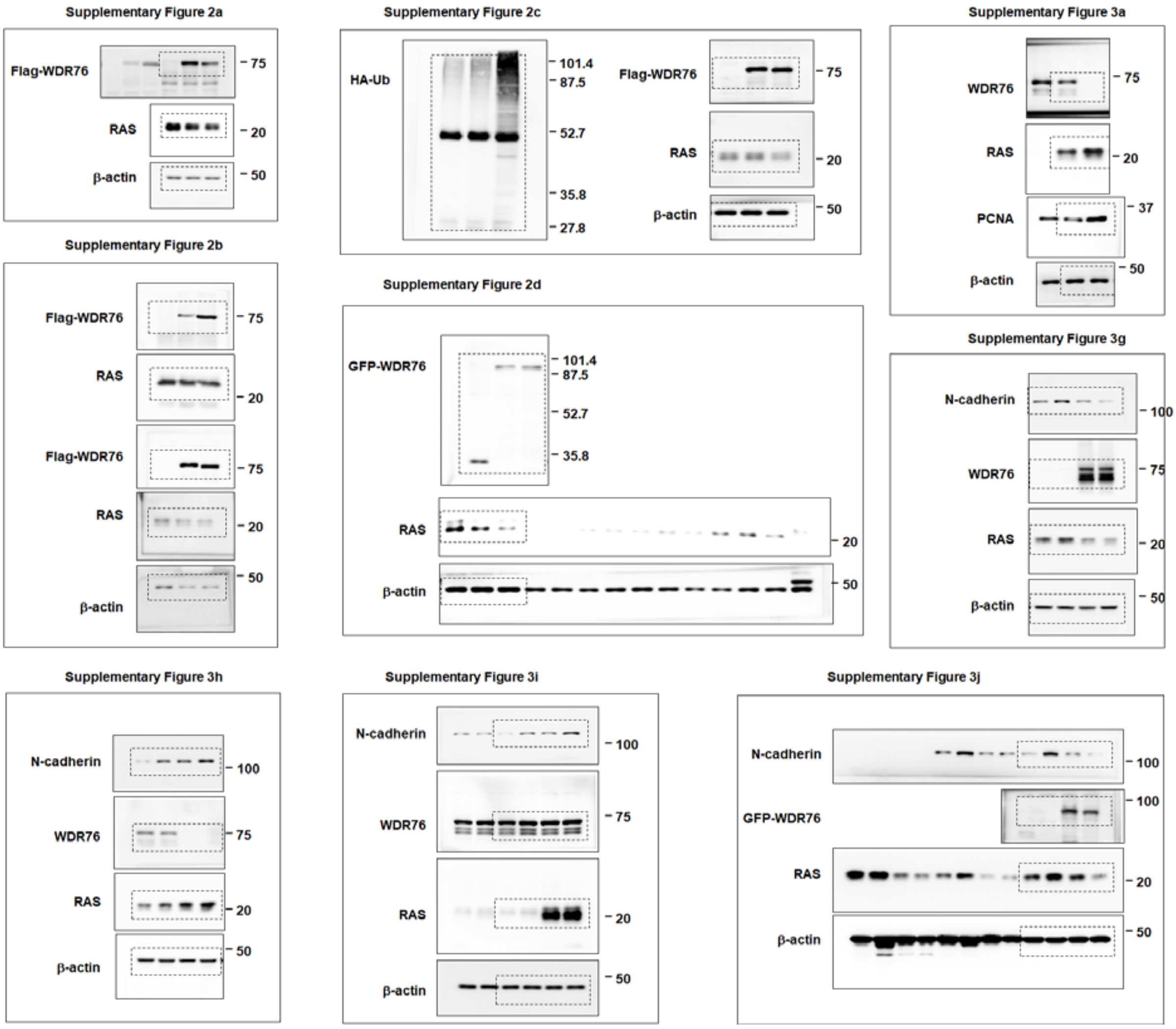

Supplementary Fig. 12 Uncropped blots for Supplementary Figure 2-3

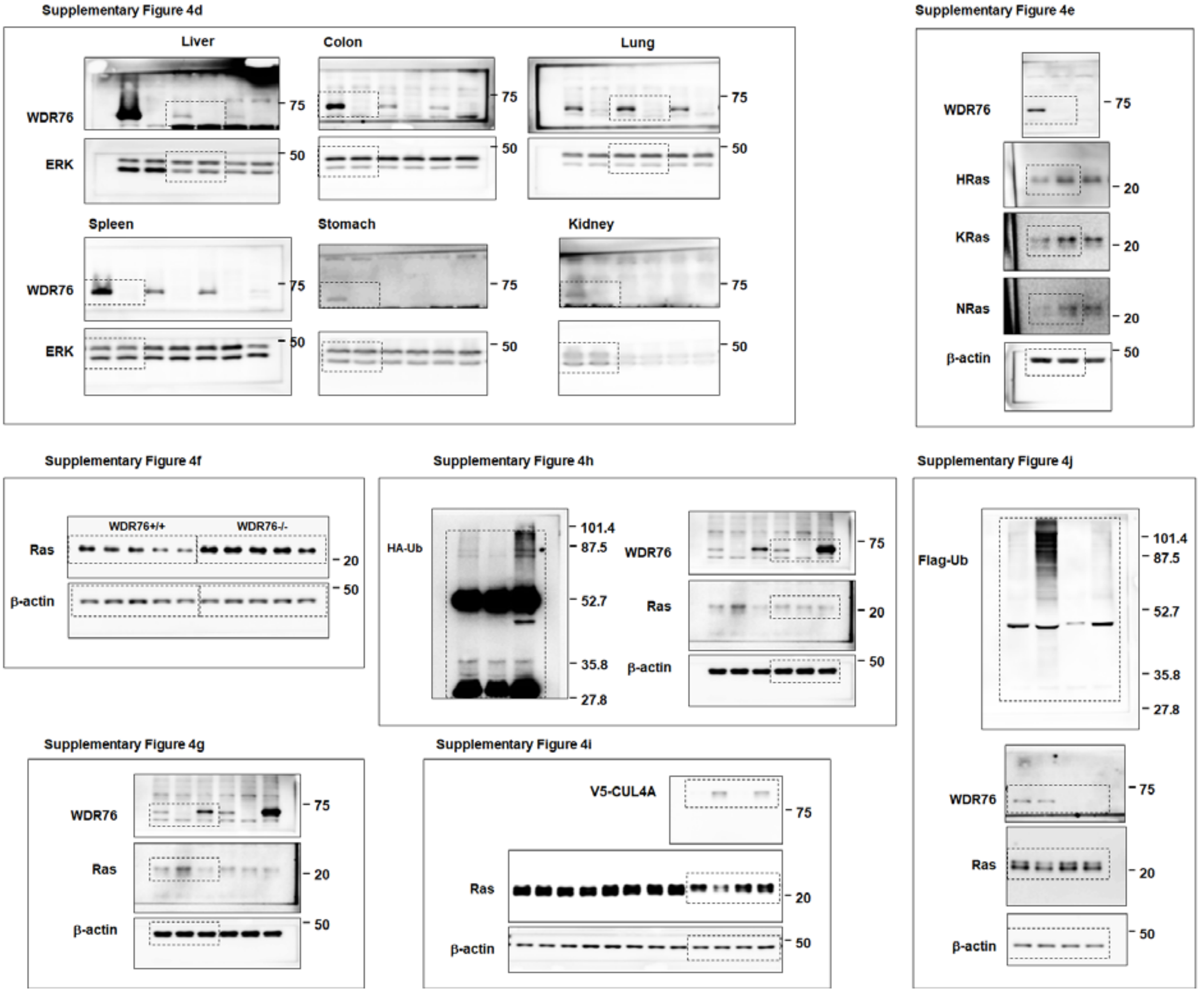

Supplementary Fig. 13 Uncropped blots for Supplementary Figure 4

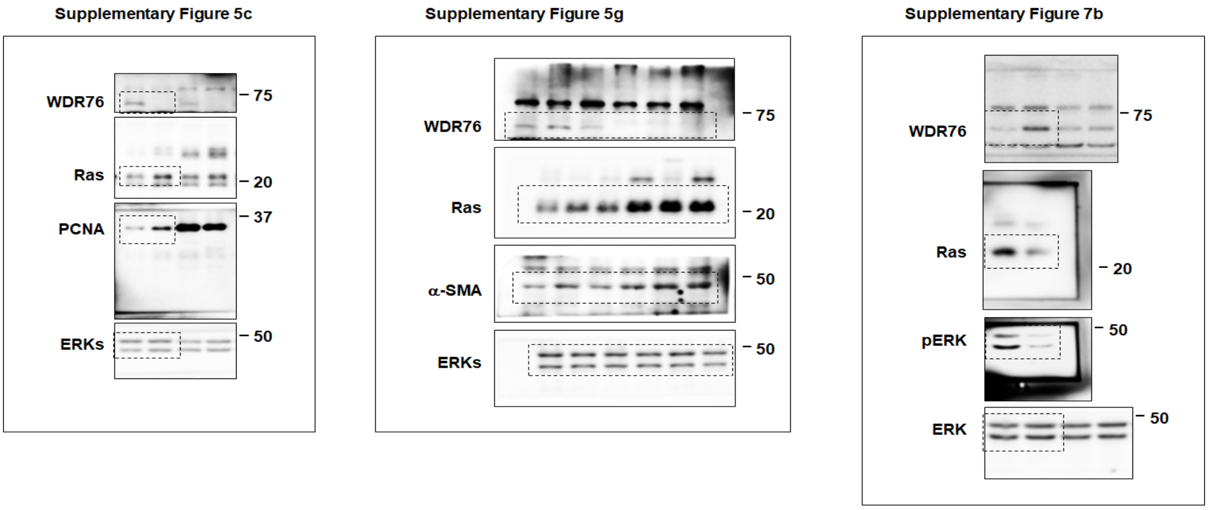

Supplementary Fig. 14 Uncropped blots for Supplementary Figure 5 and Figure 7

Supplementary Table 1 List of HRAS associated proteins

Direct association with HRAS

Protein Name	UniProtKB	Unique peptide	Sample (N/T)
JNK3	B3KQ94	K.NGVVKGQPSPSAQVQQ	N
Ras GTPase-activating protein 4	O43374	EGPLFIHRTKGK	N
GTPase Nras	P01111	R.QGVEDAFYTLVR.E<br>-.GVEDAFYTLVR.E	N/T
GTPase Kras	P01116	K.LVVVGAVGVGK.S<br>K.QAQDLAR.S	N/T
Receptor tyrosine-protein kinase erbB-2 , Neu	P04626	P.EHLGLDVPV.-	N
Tyrosine-protein kinase Fyn	P06241	X.TPRPLPVAPGSSK.T	N
Neurofibromatosis 1	P21359	K.EEFKALKTLSIFYQAGTSK.A	N
Ras GTPase-activating protein 2	Q15283	R.VSRCNQNR.L	N
Dipeptidyl peptidase 9 , DP9 , Dipeptidyl peptidase-like protein 9	Q86TI2	EFGLFEELSEGSFGWVTGIR	N
Rap1-interacting adaptor molecule	Q7Z5R6	R.RSSDTSGSPATPLK.A	N
ADP-ribosylation factor GTPase activating protein 1	B7ZBI2	R.FASAAKEGATK.F	T
Mitogen-activated protein kinase kinase kinase 6	O95382	R.AALGVLGPEVEK.E	T
Cytosolic phospholipase A2 beta	P0C869	R.FSGAPDESGAEKR.S	T
cAMP-specific 3',5'-cyclic phosphodiesterase 4D	Q08499	MASNKFK.R	T
Serine/threonine-protein kinase 38	Q15208	K.KDTGHVYAMKILR.K	T

Predictive association with HRAS

Protein Name	UniProtKB	Unique peptide	Sample (N/T)
Active breakpoint cluster region-related protein isoform b	Q12979	K.RVAEKEPINK.M	N
Rho GTPase-activating protein 10	A1A4S6	K.EQLGAVKEEK.K	N
Diacylglycerol kinase, epsilon	A1L4Q0	K.THAMMLYFSGEQTDDDISSTSDQEDIK.A	N/T
G-protein signalling modulator 3 (AGS3-like, C. elegans)	A2BFJ3	R.GWRLRDPR.K	N
Toll-like receptor 6	B2R933	R.GLIHVPKDLPLK.T	N
Heat shock 70 kDa protein 12A	O43301	R.WEGGDPGVSNQKTPTTILLTPERK.F	N
Ubiquitin conjugation factor E4 B , Ubiquitin fusion degradation protein 2	O95155	MAAMLNFNLQQLCGPKCR.D	N
Sulfotransferase 1A3/1A4	P50224	K.REIQKILEFVGR.S	N
Ras-related small GTP binding protein Rab5	P51148	M.AGRGGARRPNGPAAGNK.I	N
Gamma-actin	P63261	K.SYELPDGQVITIGNER.F	N
Keratin, type I cytoskeletal 17	Q04695	R.LSVEADINGLRR.V	N
Ankyrin repeat domain-containing protein 16	Q6P6B7	MAQPGDPRR.L	N
Rho guanine nucleotide exchange factor 18	Q6ZSZ5	R.IHFPNRAPR.R	N
Ubiquitin-protein ligase E3B	Q7Z3V4	R.LISGDNAEIDLEDLKK.H	N
Zinc finger and BTB domain-containing protein 10	Q96DT7	LKCPHCSYVAKYR	N
Ubiquitin specific protease 42	Q9H9J4	R.LTESSDGGLR.R	N
Ubiquitin specific protease 29	Q9HBJ7	K.EDMEKLNATLNTGK.E	N
Eukaryotic translation initiation factor 3 subunit L	Q9Y262	K.TVSDLIDQK.V	N
Ankyrin repeat domain-containing protein 34B	A5PLL1	R.HQSIDVK.D	T
Phospholipase C, eta 2	B9DI82	M.GAMQEGMQMVK.L	T
Cell division cycle protein 123 homolog	O75794	K.LIDKSAGGR.L	T
Integrin beta-3	P05106	K.SILYVVEEPECPK.G	T
Heat shock 70 kDa protein 1-like	P34931	R.IINEPTAAAIAYGLDK.G	T
PTPL1-associated RhoGAP 1	Q52LW3	K.RVVDHAEENKMNSK.N	T
Ankyrin repeat domain 11	Q6UB99	K.KSPFLSSAEGAVPKLDK.E	T
Ankyrin repeat domain-containing protein 23	Q86SG2	R.EALQAHVAHPRTR.C	T
Tetratricopeptide repeat domain 21A	Q8NDW8	K.EALLWYSEAMK.L	T
Tetratricopeptide repeat protein 28	Q96AY4	K.LAEQLGRREDEAK.C	T
Phosphatidylinositol 4-kinase type II	Q9BTU6	K.LELNIVPRTK.V	T
Anaphase promoting complex subunit 1	Q9H1A4	R.GAHSGVGSLQVR.W	T
Sorting nexin 14	Q9Y5W7	K.KLLKAAMK.H	T

* N: Non-Tumor, T: Tumor

Supplementary Table 2 List of Primer sequences

Primer	Forward (5'→3')	Reverse (3'→5')
WDR76 (human)	TTACGCTGTGGGGATTTTTC	GTCACCACTCTGTTACCAGTA
HectD1 (human)	ATGCTCGTGGTTATGGAAGG	TGTGTTTGTCCACTGGCATT
UBE4B (human)	GCCAGAGCAGTGAAGGAGTC	TATCACTGAGGCTCCGCTTT
USP42 (human)	GGGACTGGACCATTGAAAGA	TGAGACTGACACTGGCGAAC
UBE3B (human)	CTGTTGTGATCCAGGCCCAT	CCAGGGACACATACCACACC
USP29 (human)	CACTGAGTCCACCAATGGCT	GCTCCCGATATGGCTGACAA
β-actin (human)	AATCTGGCACCACACCTTCTAC	ATAGCACAGCCTGGATAGCAAC
GAPDH (human)	GTCTCCTCTGACTTCAACAGCG	ACCACCCTGTTGCTGTAGCCAA
HPRT (human)	CCCTGGCGTCGTGATTAGTG	TCGAGCAAGACGTTCAGTCC
HRAS (human)	GGAAGCAGGTGGTCATTG	AGACTTGGTGTTGTTGATGG
KRAS (human)	AAACAGGCTCAGGACTTAG	GTATAGAAGGCATCATCAACAC
NRAS (human)	AAGAGTTACGGGATTCCATTC	CCATCATCACTGCTGTTGA
WDR76 (mouse)	GTCACTGGGAGGTGGCTAAC	GAAGACTTCCACCCGTCGTG
HRas (mouse)	ATCATCTGCCTTGCTCCT	GACTATCCTTGTGTTGCCTAT
KRas (mouse)	CTGGTAGGGAATAAGTGTGATTT	TGTATAGAAGGCATCGTCAAC
NRas (mouse)	GCTGGTGTGAAATGACTGA	TCGGTAAGAATCCTCTATGGT
TNF-α (mouse)	GCTCCCCAAGAAGACAGGG	GATAGCAAATCGGCTGACGG
IL-1β (mouse)	AATGCCACCTTTTGACAGTGATG	TGGAAGGTCCACGGGAAAGA
β-actin (mouse)	ATCATTGCTCCTCCTGAGCG	ACGCAGCTCAGTAACAGTCC
GAPDH (mouse)	ACCACAGTCCATGCCATCAC	TCCACCACCCTGTTGCTGTA
HPRT (mouse)	CAGTCCCAGCGTCGTGATTA	TGGCCTCCCATCTCCTTCAT
