# Supplementary material for: WDR76 is a RAS binding protein that functions as a tumor suppressor via RAS degradation
Source: Nat Commun. 2019 Jan 17;10:295. doi: 10.1038/s41467-018-08230-6 (PMC6336889; doi:10.1038/s41467-018-08230-6)
Supplement: Supplementary file 3 — Reporting Summary [file 41467_2018_8230_MOESM3_ESM.pdf]

nature research

Corresponding author(s): NCOMMS-18-15599C

Last updated by author(s): Dec 9, 2018

Reporting Summary

Nature Research wishes to improve the reproducibility of the work that we publish. This form provides structure for consistency and transparency in reporting. For further information on Nature Research policies, see Authors & Referees and the Editorial Policy Checklist.

Statistics

For all statistical analyses, confirm that the following items are present in the figure legend, table legend, main text, or Methods section.

n/a	Confirmed	
☐	☒	The exact sample size (*n*) for each experimental group/condition, given as a discrete number and unit of measurement
☐	☒	A statement on whether measurements were taken from distinct samples or whether the same sample was measured repeatedly
☐	☒	The statistical test(s) used AND whether they are one- or two-sided<br>*Only common tests should be described solely by name; describe more complex techniques in the Methods section.*
☒	☐	A description of all covariates tested
☐	☒	A description of any assumptions or corrections, such as tests of normality and adjustment for multiple comparisons
☐	☒	A full description of the statistical parameters including central tendency (e.g. means) or other basic estimates (e.g. regression coefficient) AND variation (e.g. standard deviation) or associated estimates of uncertainty (e.g. confidence intervals)
☐	☒	For null hypothesis testing, the test statistic (e.g. *F*, *t*, *r*) with confidence intervals, effect sizes, degrees of freedom and *P* value noted<br>*Give P values as exact values whenever suitable.*
☒	☐	For Bayesian analysis, information on the choice of priors and Markov chain Monte Carlo settings
☒	☐	For hierarchical and complex designs, identification of the appropriate level for tests and full reporting of outcomes
☐	☒	Estimates of effect sizes (e.g. Cohen's *d*, Pearson's *r*), indicating how they were calculated

Our web collection on statistics for biologists contains articles on many of the points above.

Software and code

Policy information about availability of computer code

Data collection	No software was used to collect data.
Data analysis	Prism software (GraphPad) was used to generate graphs and perform statistical analyses.<br>MultiGauge software (Fuji) was used for western blot analyses.<br>LSM Image Browser (Zeiss) sofware was used for immunofluorescence confocal microscopy analysis.

For manuscripts utilizing custom algorithms or software that are central to the research but not yet described in published literature, software must be made available to editors/reviewers. We strongly encourage code deposition in a community repository (e.g. GitHub). See the Nature Research guidelines for submitting code & software for further information.

Data

Policy information about availability of data

All manuscripts must include a data availability statement. This statement should provide the following information, where applicable:

- Accession codes, unique identifiers, or web links for publicly available datasets
- A list of figures that have associated raw data
- A description of any restrictions on data availability

The data that support the findings of this study are available from the corresponding author upon reasonable request.

Field-specific reporting

Please select the one below that is the best fit for your research. If you are not sure, read the appropriate sections before making your selection.

☒ Life sciences ☐ Behavioural & social sciences ☐ Ecological, evolutionary & environmental sciences

For a reference copy of the document with all sections, see nature.com/documents/nr-reporting-summary-flat.pdf

Life sciences study design

All studies must disclose on these points even when the disclosure is negative.

Sample size	Experiments were performed at least three independent times. Sample size in mice experiments were estimated based on previous published experiments. At least three or more mice were used in each experiment to obtain statistical analysis. Exact numbers of animals used in individual experiments are indicated in the figure legends. In this study, the statistic analysis was obtained using student t test and the values represents means plus minus standard deviation or standard error of the mean.
Data exclusions	No data were excluded.
Replication	All experiments were reliably reproduced and results are presented as mean +/- SD or SEM as indicated in the figure legends. The results presented have been successfully replicated in at least three independent experiments, with sufficient independent samples.
Randomization	All mice were age and sex-matched (male mice) and then randomized into the different groups.
Blinding	The investigators were not blinded to group allocation during experiments. Conclusions were made based on quantitative parameters and statistical significance of the data, and thus on experimental observations, independent of blinding.

Reporting for specific materials, systems and methods

We require information from authors about some types of materials, experimental systems and methods used in many studies. Here, indicate whether each material, system or method listed is relevant to your study. If you are not sure if a list item applies to your research, read the appropriate section before selecting a response.

Materials & experimental systems

n/a	Involved in the study	
☐	☒	Antibodies
☐	☒	Eukaryotic cell lines
☒	☐	Palaeontology
☐	☒	Animals and other organisms
☒	☐	Human research participants
☒	☐	Clinical data

Methods

n/a	Involved in the study	
☒	☐	ChIP-seq
☒	☐	Flow cytometry
☒	☐	MRI-based neuroimaging

Antibodies

Antibodies used	anti-Ras (Millipore, 05-516), anti-HRas (Santa Cruz Biotechnology, sc-520), anti-KRas (Santa Cruz Biotechnology, sc-30), anti-NRas (Santa Cruz Biotechnology, sc-31), anti-p-ERK (Cell Signaling Technology, #9101S), anti-ERK (Santa Cruz Biotechnology, sc-514302), anti-PCNA (Santa Cruz Biotechnology, sc-56), anti-N-cadherin (BD Bioscience, #610920), anti-aSMA (Abcam, ab7817), anti-Myc (Cell Signaling Technology, #2276S), anti-FLAG (Sigma-Aldrich, F7425), anti-HA (Santa Cruz Biotechnology, sc-7392), anti-V5 (MBL International., M167-3), anti-GFP (Santa Cruz Biotechnology, sc-8334), anti-GST (Santa Cruz Biotechnology, sc-374171), anti-Ki67 (Abcam, ab15580), anti-aSMA (Abcam, ab7817), and anti-b-actin (Santa Cruz Biotechnology, sc-47778). WDR76 polyclonal antibody was generated from immunization of rabbits with partially purified WDR76 proteins (GST-WDR76 1-300; Abfrontier, Korea)
Validation	All these antibodies were validated by manufacturers and largely described in the literature.<br>WDR76 antibody was validated using WDR76 KO mice.

Eukaryotic cell lines

Policy information about cell lines

Cell line source(s)	All cells (SK-Hep1, Huh7, HepG2, PLC/PRF/5, and Hep3B, Lovo, T24T, HEK 293, and HEK293T) were obtained from the American Type Culture Collection. WDR76+/+ or WDR76-/- MEF cells were prepared from E13.5 mouse embryos.
Authentication	SK-Hep1 cells were authenticated by Cosmogenetech (Daejeon, Korea)

Mycoplasma contamination	Negative
Commonly misidentified lines (See ICLAC register)	None

Animals and other organisms

Policy information about studies involving animals; ARRIVE guidelines recommended for reporting animal research

Laboratory animals	The ES cell clone (IST11346A1) for WDR76 knockout were obtained from KOMP Repository and injected into blastocysts from C57BL/6 mice to derive chimera mice by Yonsei laboratory Animal Research Center (Seoul, Korea). Plasmid DNA of pCB-WDR76 was used for generation of Cre-inducible WDR76 transgenic mice. Transgenic mice were produced, including linearization of DNA and microinjection, by Macrogen (Seoul, Korea). WDR76 Tg mice were obtained and transgene expression was examined by crossing with albumin-CRE mouse strain (obtained from the Jackson Laboratory). HRasG12V mice were obtained from Consejo Superior de Investigaciones Científicas (CSIC). All mice are in the C57BL/6 background.
Wild animals	The study does not involve wild animals.
Field-collected samples	The study did not involve samples collected from the fields.
Ethics oversight	All animal experiments were performed in accordance with the Korean Food and Drug Administration guidelines. Protocols were reviewed and approved by the Institutional Animal Care and Use Committee (IACUC) of Yonsei University.

Note that full information on the approval of the study protocol must also be provided in the manuscript.
